# Habilitation of sleep problems among mothers and their children with autism spectrum disorder: Insights from multi-level exploratory dyadic analyses

**DOI:** 10.3389/fresc.2022.915060

**Published:** 2022-09-21

**Authors:** Wasmiah Bin Eid, Mengyu Lim, Giulio Gabrieli, Melanie Kölbel, Elizabeth Halstead, Gianluca Esposito, Dagmara Dimitriou

**Affiliations:** ^1^ Sleep Education and Research Laboratory, UCL Institute of Education, London, UK; ^2^ Psychology Program, School of Social Sciences, Nanyang Technological University, Singapore; ^3^ Department of Developmental Neurosciences Unit, UCL Great Ormond Street Institute of Child Health, London, UK; ^4^ Affiliative Behaviour and Physiology Lab, Department of Psychology and Cognitive Science, University of Trento, Italy

**Keywords:** autism, ASD, sleep, maternal sleep, child sleep, cortisol, actigraphy

## Abstract

Few habilitation strategies for children with autism spectrum disorder (ASD) consider their sleep-related problems. Together with the fact that caregivers of children with ASD also face issues with sleep, there may be yet-to-be uncovered relationships between caregiver-child sleep patterns and sleep quality, offering a key opportunity for clinicians to consider the needs of both child and caregiver in terms of sleep. 29 dyads of mothers and their children with ASD were recruited for this cohort study and both subjective (self-report questionnaires and sleep diaries) and objective (cortisol samples and actigraphy) measures of sleep were collected to investigate significant predictors of sleep quality. Comparative, correlational, and hierarchical analyses were conducted. Findings indicated that both mother and child experience sleep deprivation in terms of shorter sleep duration and poor sleep quality in terms of longer sleep onset latencies and a higher frequency of wake bouts. Exploratory hierarchical analyses also found that child-related sleep difficulties such as sleep disordered breathing and night waking significantly predict mothers’ sleep quality, which may point to the bi-directional influence of mother-child sleep. Based on these findings, it is recommended that clinicians adopt a family systems perspective and consider the sleep environment of the household, particularly that of the caregiver and child, when designing interventions for sleep-related problems in ASD. Finally, there is a need for additional support to promote good quality sleep among caregivers of children with ASD to bolster out-of-clinic care.

## 1. Introduction

Currently, in the habilitation of children with Autism Spectrum Disorder (ASD), an integration of pharmacological, behavioural, sociopsychological and educational approaches is recommended (1). However, it is well-known that children with Autism Spectrum Disorder (ASD) also face problems with sleep—up to 80% of these children face issues with sleep, regardless of their actual severity of ASD (2). The high prevalence of sleep problems in children with ASD hint at the need for habilitation guidelines for these children to include sleep-related perspectives (3), although there is still a lack of literature in this area. Furthermore, as a long-term developmental condition that requires a longitudinal view of habilitation at each stage of life, the lack of a developmental perspective regarding sleep among children with ASD is also concerning. Hodge and colleagues (4) recommends a further segmentation of children with ASD by age groups so that developmental effects are not concealed. A related longitudinal study by Gregory and O’Connor (5) found that the prevalence of sleep problems among typically developing children aged 4 to 15 years tended to decrease over time. Other studies have also reported similar findings (6–8). A possible explanation for this phenomenon lies in the implementation of national education policies having a beneficial effect on sleep problems by enforcing more regular sleep schedules, although it is still unknown if this is true of children with ASD. With more recent studies, findings emphasising the importance of addressing the research gap of sleep problems among children with ASD, and particularly the developmental perspective of these problems have surfaced: sleep problems eventually contribute to worsened behavioural problems in the day (2). Conversely, individuals with ASD but fewer sleep problems have more positive outcomes in terms of social interactions and affect (9, 10). The findings of these studies suggest that treatment of sleep problems in ASD is beneficial both towards the general improvement of the individual’s rest and well-being, but also more specifically in alleviating the symptoms of the developmental disorder. There are therefore real implications of sleep in the treatment course and habilitation of children with ASD in their daily lives.

When considering ASD habilitation, caregivers must not be neglected. As the main source of caregiving, well-being advocacy and coordinator of treatment plans for children with ASD outside of professional healthcare settings, parents of these children face the tremendous responsibility of ensuring treatment continuity and adherence at home (11). Despite the historical misattribution of the causes of ASD to the “refrigerator mother” and poor quality of care (12, 13), the success of habilitation for children with ASD depends greatly on parental involvement. In fact, habilitation recommendations for children with ASD often also include parental involvement and support groups for parents (1). The inclusion of the parents or the main caregiver (often the mother) adds another degree of complexity when considering the habilitation of children with ASD. This is because bi-directional interactions between caregiver and child (14), together with the added disability of the child due to the presence of ASD, contribute to a long-term vicious cycle of poorer quality of caregiving and worse outcomes for both caregiver and child overall (15–17). Some of these negative caregiver outcomes have been previously uncovered (18–21), especially in the domains of stress (22) and physical ailments (23–26). These effects have been observed in the body in terms of endocrine and immune changes (27, 24, 28–30). Additionally, similar to their children with ASD, caregivers also experience greater incidences of sleep problems, which may be related to disturbances in endocrine and immune processes mentioned above. Based on a study by Gallagher and colleagues (30), caregivers of children with developmental disabilities (including ASD) report more problems in terms of increased sleep latency, poorer sleep efficiency, and decreased sleep duration and quality compared to control participants of the same age group. Based on clinical cut-offs, the sleep difficulties described here qualify these caregivers to be categorized as poor sleepers, a finding that is supported by another similar study (31). In qualitative studies, sleep problems have also commonly surfaced as one of the most challenging issues in caring for children with ASD (23, 32, 33). These sleep problems have then been found to be predictive of increased health risks (34–37), which may have contributed to the reports of physical maladies and physiological changes in caregivers. Cortisol is one such hormone that can be used to study physiological changes associated with sleep problems. Although commonly studied as a marker of stress (29), cortisol also has a diurnal “U”-shaped pattern of secretion in healthy adults from daytime (i.e., reaching minimum levels at night and peaking again the following morning) that can be affected by poor sleep. For example, our previous study found that mothers of children with ASD with flattened cortisol profiles (i.e., below median ratio of daytime cortisol with respect to nighttime cortisol) report significantly poorer sleep, even after controlling for their child’s age, sex, and severity of ASD (38).

Thus far, the relationships between ASD, child sleep, and caregiver sleep have not yet been extensively explored. Although tangential, several studies have examined caregiver-child synchrony or attunement, which involves analyzing the patterns of commonality in behavioural and physiological signals between caregiver and child (39–41). In their papers, the authors reported that synchronicity between caregiver and child have significant relationships to caregivers’ attachment anxiety (40), as well as parenting stress (41), where reduced reduced synchrony between mother and child was correlated with increased parenting stress. Albeit most of this research is related to typically developing children, it is also of interest here since a previous study by the research team has found significant correlations between mothers’ cortisol profiles and the sleep quality of their children with ASD (38), suggesting the presence of a dyadic effect in mother-child sleep in the context of ASD. However, due to the lack of normative data for cortisol measurements in the past study, other sources of data such as actigraphy could be considered as a further extension in this area. Actigraphy is a commonly used non-invasive objective measure of sleep (42) that tracks periods of activity and rest over a period of days or weeks using an accelerometer. Due to its size and cost effectiveness, actigraphy is most useful for longitudinal, naturalistic research on sleep-wake patterns (43). More generally, studies on sleep quality in caregivers and their children with ASD often make use of only subjective measures such as self-report questionnaires or sleep diaries (e.g., (23, 31, 32, 30, 33)). This highlights a more generalized need for objective measures of sleep quality in both caregiver and child.

### 1.1. Aim of current study

Children with ASD require support from all levels of society. In the habilitation of these children and in supporting their main caregivers, there is a significant research gap addressing their needs in the context of sleep, which would have great impact on treatment outcomes and the capacity of their caregivers to continue to provide quality care outside of the professional healthcare settings. Additionally, there is still a lack of understanding of how caregiver and child sleep may be mutually related to each other. Therefore, the present study aims to extend upon the findings in our previous study (38), by using a combination of both subjective (i.e., self-reported questionnaires and sleep diaries) and objective (i.e., actigraphy and cortisol) measures, in both caregivers and their children with ASD. In this case, mothers as the typical main caregivers of children with ASD were selected. Children with ASD aged between 6 and 16 years of age were included in this study to provide insight on potential developmental trajectories of sleep problems, addressing another prominent research gap. In line with potential schooling milestones in contributing to different wake-sleep cycles at various stages of life, these children are segmented into school-going children aged 6 to 13 and adolescents aged 14 to 16. In the UK where this study was conducted, these age categories represent Years 2 to 9, and Years 10 and 11 (years of preparing for national examinations) respectively. The present study shall explore, using both mother- and child-related variables (the full list of questionnaire, behavioural and physiological variables are described in subsequent sections), significant predictors of sleep quality in both mothers and their children with ASD. Additionally, mother-child sleep data were also collated and analysed in dyads as an exploration of the interpersonal effects of mother-child sleep. Findings were then subsequently discussed in terms of their clinical implications and potential recommendations for the habilitation of children with ASD in a family or household setting.

## 2. Methodology

The current study employs a cohort design with mothers and their children with ASD. The study, materials and procedure were approved by the UCL Research Ethics Committee (approval 16682/001) and conducted in accordance with the Declaration of Helsinki. Data are not publicly accessible but can be made available on request to D.D.

### 2.1. Participants

29 dyads consisting of mothers and their children diagnosed with ASD (i.e., N=58) were recruited from the London area. Recruitment was conducted via snowball sampling through online advertisements and ASD-related events and workshops (44) and lasted from May to October 2019. Mothers had to be above 18 years of age and must be currently caring for a child aged between 6 and 16 years. The child must be clinically diagnosed with ASD (i.e., by a general practitioner, paediatrician or other health professional). Both mother and child must be living together at home. Participants were excluded if they were also caring for another individual (e.g., child, parent, partner, other family member, or friend) with a chronic illness or other developmental disorders. Participants were also excluded if they were experiencing other acute stressors such as divorce or bereavement within the last 12 months from the point of recruitment in the study.

### 2.2. Questionnaires

Self-report questionnaires (CARS-2; (45) and CSHQ (46) were administered. Additionally, a demographic questionnaire asking the mothers’ age and ethnic heritage, as well as their child’s age and sex was also administered. Mothers also reported their current employment position. All questionnaires were completed by the mothers.

#### 2.2.1. Childhood autism rating scale-second edition (CARS-2)

CARS-2 (45) measures child ASD severity based on behavioural items by focusing on dimensions such as the child’s adjustment to change, affective response, imitation, interpersonal relations, use of body and objects, and visual and listening responses. It contains 15 items rated on a 4-point Likert scale, where lower scores refer to age-appropriate behaviour and higher scores indicate atypical behaviour. These scores are then summed to form a total CARS-2 score ranging from 15 to 60, where 15 to 30 points mean no or minimal signs of ASD behaviour, 30 to 36 points indicate mild or moderate signs of ASD behaviour, and 37 to 60 points indicate strong signs of ASD behaviour.

#### 2.2.2. Child’s sleep habits questionnaire (CSHQ)

CSHQ (46) is a subjective measure of the child’s sleep problems (e.g., sleep anxiety and night wakings) and habitual sleep behaviour (e.g., sleep duration, bedtime and sleep onset behaviour). The 45 items on CHSQ are rated on a 3-point scale indicating frequency where “1” refers to rarely (i.e., never or only once within the past week), “2” refers to sometimes (i.e., 2 to 4 times within the past week) and “3” refers to usually (i.e., 5 to 7 times within the past week). Theoretically, CSHQ scores range from 33 to 99, where a score above 41 indicates a paediatric sleep disorder. For each subscale’s interpretation, normative clinical data from Owens and colleagues (46) were used to determine clinically significant cut-off scores, where scores above the clinical mean indicated sleep difficulties in the following categories: bedtime resistance (>9.43), daytime sleepiness (>11.99), night waking (>5.69), parasomnias (>11.22), sleep anxiety (>7.09), sleep disordered breathing (>4.71), sleep duration (>4.94), and sleep onset (>1.80).

### 2.3. Actigraphy and sleep diaries

The MotionWatch8 is a medical device approved by the US Food and Drink Administration (K132764) which can monitor day and night-time movement activity by using an internal accelerometer with a sampling rate of 30 s (i.e., 1 epochs; CamNtech Ltd, 2021). Mother and child wore the watch for a minimum of 5 consecutive nights on the non-dominant wrist, at the same time. Although actigraphy provides automated sleep onset estimates, this data might be less accurate if the person was not moving but, for instance, was watching the television. Additionally, typical social events such as family gatherings, celebrations or sleepovers may occur during the period of data collection, which may affect the accuracy of actigraphy readings if the context of participants’ daily living was not known (43).

Therefore, two sleep diaries were also created (one for maternal sleep and one for child sleep) to provide an additional source of data to improve data accuracy. The sleep diaries were given to participants in hard copy and included standard questions on sleep parameters such as time when lights are put out, time of getting out of bed, and estimated total sleep time (please see Table 1 for more details). Mothers were asked to note if there were any unusual events such as sleepovers, family birthdays and child being unwell in the sleep diary. Sleep diaries were completed on the same nights as when the actigraphy data collection took place. Extracted data of interest in this study in terms of sleep quantity and quality are summarised in Table 1 below. As far as possible, the source of data (i.e., actigraphy, sleep diary, or a calculation derived from both sources of data) and calculation formulae are also provided.

**Table 1:** Sleep variables measured by actigraphy and sleep diary data.

Sleep variable category	Actigraphy data	Sleep diary data
Sleep quantity (i.e., duration and timings of sleep and sleep-related activities)	Actual sleep time: actual total time spent asleep, calculated by wake/sleep categorisation(ˆ,${}^{*}$)	Assumed Sleep Time: assumed/estimated total time spent asleep
	Fell asleep: time of falling asleep, recorded by first sleep categorisation	Lights out: time of turning off lights in the bedroom
	Waketime: time of waking up, recorded by last wake categorisation	Get up: time of getting out of bed
		Time in bed: total time spent in bed; calculated by get up – lights out
Sleep quality (i.e., presence and severity of disturbed sleep)	Wake bout duration: average length of each wake bout in minutes	
	Wake bout frequency: number of contiguous wake sections in the epoch-by-epoch wake/sleep categorisationˆ	
	Fragmentation index: degree of movement during the night and an indication of sleep quality. Fragmentation index is calculated by summing the percentage of wake epochs of within the sleep period and the percentage of sleep stages of one minute out of all sleep stages (47, 48)ˆ	

Note that “∧” indicates that the mothers’ data were subsequently used as a dependent variable in hierarchical analyses, while “${}^{*}$” indicates that the children’s data were used as a dependent variable in hierarchical analyses. Descriptive and comparative results from all other variables are reported in Section 3.2. Some of the other variables are also incorporated as predictors in hierarchical analyses in Section 3.3.

Actigraphy data were analyzed using the CamNtech Motionware computer software. The sleep diaries then provided the assumed sleep time data, lights out data and information for calculations of sleep efficiency and sleep onset latency, and also served to verify that the sleep times identified by actigraphy were accurate. Based on raw data extracted from actigraphy and sleep diaries (Table 1), two other variables can be calculated. The first, Sleep Onset Latency, refers to the time taken to fall asleep and is calculated using the time elapsed between Lights Out and Fell Asleep. The second, Sleep Effiency, is expressed as a percentage of Actual Sleep Time out of Time in Bed.

### 2.4. Cortisol salivary samples

Oral fluid collectors (OFC) from Soma Bioscience Ltd. (Wallingford, UK) were used to collect salivary samples from each mother. The OFC consists of a synthetic polymer swab that was designed and mixed with a buffer. OFC-stored samples can be stored at room temperature for a long period of several weeks and are not affected by the presence of recent food and drink ingestion. To determine the salivary assay range for cortisol (0.25–32.0 ng/ml), an enzyme immunoassay test kit was used (Soma Bioscience Ltd., Wallingford, UK). Mothers were asked to take three samples of their saliva to be stored in the OFC. Each OFC was labelled with unique ID numbers corresponding to the mothers’ participant IDs and also colour coded to indicate the time of expected collection. The first sample was taken within 30 min of mothers’ awakening. The second sample was taken in the afternoon at 4pm, and finally the third sample was taken before habitual sleep. Cortisol profiles were assessed using all 3 samples. Numerical values in terms of raw morning cortisol and diurnal ratio (DCR; PM (bedtime)/AM) are subsequently reported. Typically, healthy diurnal cortisol expression follows a U-shaped curve over the course of a day. Therefore, the three samples were also used to identify participants with a “flattened” cortisol expression pattern by using the median split method (below median ratio of morning cortisol with respect to bedtime cortisol, and below median ratio of afternoon cortisol with respect to bedtime cortisol).

### 2.5. Procedure

Data collection began after recruitment from May to October 2019. The study lasted for a maximum of 7 days. On day 1, research team members met with the mothers of the study and provided a study materials pack containing study information and accompanying instructions for each component of the following materials: informed consent forms, self-report questionnaire measures, sleep diaries, actigraphy devices and salivary collectors for cortisol samples for each mother. Verbal informed consent was obtained from the child and written informed consent from their parent or legal guardian. Upon obtaining informed consent, the mothers and children were asked to monitor their sleep with the actigraphy for one week (at least 5 consecutive days) and fill in sleep diary for themselves and their child, which are used to support the analysis of actigraphy data (49). The questionnaires were completed within the first 2 days of the study. Cortisol samples were obtained at the three different time points as described above, on either day 2, 3, or 4. At the midpoint of the study on day 3, research team members called each participant to ask if they had collected a salivary sample, and if there were any questions or discomforts of wearing a watch. On day 7, participants were contacted again to arrange for a suitable date to retrieve the study materials (i.e., completed questionnaires, salivary samples and actigraphy devices).

### 2.6. Analytic plan

The analytic plan consisted of four main approaches: descriptive statistics, correlation between cortisol samples and actigraphy data, hierarchical multiple regression to understand predictors of various sleep behaviours, and dyadic analysis of mother and child sleep variables. A majority of the statistical analyses were performed on SPSS® version 26 (IBM Corporation, Armonk, NY, USA) for Mac®, while correlational analyses were conducted on Python (v. 3.5.8) on Linux (Kernel 5.4.0-73-generic). In comparative descriptive analyses, data were assessed for the assumptions of normality and homogeneity of variance using Shapiro-Wilk test to determine if parametric tests were appropriate. Otherwise, where the assumptions were not fulfilled, non-parametric tests were used instead. Exploratory hierarchical multiple regression analyses was used to identify variables that predicted sleep behaviour in both mother and child, and cortisol value in mothers. Additionally, in the final approach, mothers’ and children’s Actual Sleep Time were evaluated. Mother-child dyads were then classified into either synchronous-below median “Below Median” (i.e., both mother and child Actual Sleep Time below median values of their respective samples), synchronous-above median “Above Median” (i.e., both mother and child Actual Sleep Time above median values of their respective samples), or unmatched “Unmatched” (i.e., either only the mother or child Actual Sleep Time was below the median value of their respective samples and vice versa). From there, F-tests were conducted between groups to uncover if synchronicity between mother and child in terms of Actual Sleep Time had an effect on the mother’s and the child’s sleep behaviours. Additionally, the absolute difference in Actual Sleep Time between mother and child were also correlated with mother and child sleep variables to determine if the difference in quantity of sleep between mother and child were significantly related to discrete sleep behaviours. Finally, dyads were classified into whether the mother or the child had longer Actual Sleep Times. Independent samples t-tests were conducted with this grouping to determine if there are significant differences in discrete sleep behaviours if one member of the dyad slept for longer than the other.

## 3. Results

### 3.1. Participant characteristics

Participant characteristics are summarised in Table 2.

**Table 2:** Participant characteristics.

Participant characteristics	Distribution (N=29 dyads)
*Overall characteristics*	
Heritage	White (n=19)
	Asian (n=8)
	Black (n=2)
*Mother characteristics*	
Age of mother	mean=42, SD=7.72, range=26 to 61
Employment	Full-time (n=4)
	Part-time (n=10)
	Stay-at-home (n=15)
*Child characteristics*	
Age of child	mean=10, SD=3.41, range=6 to 18
School-aged children (n=21):	mean=9.29, SD=2.26, range=6 to 13
Adolescents (n=8):	mean=15.25, SD=1.58, range=14 to 18
Sex of child	5 female
	24 male
CARS-2 score	mean=39, SD=5.23, range=30 to 51.5

### 3.2. Descriptive and comparative actigraphy data

Objective sleep profiles of mothers and their children with ASD over 5 consecutive days were assessed using actigraphy. Analysis of the actigraphy data was verified by the sleep diaries for the sleep onset parameter, particularly as actigraphy has been found to overestimate sleep onset latencies (50). Actigraphy data showed that mothers fell asleep on average 1h and 31 min later than their children (Table 3). However, children took about 38 min longer to fall asleep at night compared to their mothers, who on average fell asleep within 22 min. Both groups experienced similar levels of sleep fragmentation at night (26.8% in mothers and 25.7% in children) and both showed low sleep efficiency (79.6% in mothers and 71.5% in children), although children’s sleep efficiency is significantly lower than their mothers’.

**Table 3:** Sleep profiles of mothers and children based on actigraphy and sleep diary data.

Actigraphy variable	Mother	Mother 95%CI	Child	Child 95%CI	p-value	Effect size
	Mean (SD) [Range]		Mean (SD) [Range]			
Assumed sleep time	07:34 (00:58) [05:01–09:28]	[07:13,07:56]	08:13(01:03) [05:22–10:16]	[07:51,08:36]	.02${}^{*}$	−.91
Actual sleep time	06:31 (00:58) [03:53–08:48]	[06:08,06:53]	07:06 (00:52) [04:58–09:20]	[06:45,07:26]	.21	.63
Sleep efficiency (%)	79.58 (6.25) [68.80–89.90]	[77.21,81.96]	71.51 (14.05) [28.75–86.50]	[66.17,76.86]	.02∗,^ˆ	.68
Lights out	23:37 (01:20) [21:17–03:34]	[21:18,23:04]	21:32 (01:09) [19:25–23:22]	[21:06,21:59]	<.001${}^{***}$	−1.66
Fell asleep	00:02 (01:14) [22:03–03:46]	[23:33,00:30]	22:31 (01:12) [19:49–01:18]	[22:02,22:58]	<.001${}^{***}$	−1.24
Sleep onset latency (min)	22 (12) [10–53]	[17,27]	60 (42) [3–149]	[44,77]	<.001∗∗∗,^ˆ	1.23
Waketime	07:23 (01:02) [06:10–10:49]	[06:59,07:46]	06:59 (00:45) [05:51–08:52]	[06:42,07:16]	.17ˆ	.37
Get up	07:33 (01:03) [06:20–10:50]	[07:09,07:57]	07:20 (00:44) [05:51–09:18]	[07:03,07:37]	.84ˆ	.05
Time in Bed	07:59 (00:58) [05:11–09:50]	[07:36,08:21]	09:34 (01:06) [07:29–12:34]	[09:09,09:59]	<.001${}^{***}$	1.52
Wake bout duration (min)	4 (7) [0–40]	[1,7]	2 (2) [0–16]	[1,3]	.03∗,^ˆ	.59
Wake bout frequency (no.)	37.23 (14.79) [17.80–85.10]	[31.69,42.86]	38.39 (11.62) [17.00–67.80]	[33.97,42.81]	.41ˆ	.22
Fragmentation index (%)	26.81 (8.92) [13.70–47.60]	[23.41,30.20]	25.70 (7.52) [13.40–40.70]	[22.84,28.56]	.61	−.13

Unless indicated otherwise, all units are in terms of time (HH:MM). Note that “∧” indicates non-parametric test used in the comparison between mother and child data, while “${}^{*}$” indicates statistical significance.

When analysing this data between the different age groups of children with ASD (school-aged and adolescents; Table 4), school-aged children fall asleep significantly earlier (average sleep onset at 2207, SD=1 h 6 min) compared to the adolescents (average sleep onset at 2,322, SD=47 min). Nevertheless, both groups sleep less than the recommendations for sleep duration by the National Sleep Foundation, which range from 9 to 11 h for school-aged children and 8 to 10 h for adolescents (51).

**Table 4:** Sleep profiles of children according to their age group based on actigraphy and sleep diary data.

Actigraphy variable	School-aged children	School-aged children 95%CI	Adolescents	Adolescents 95%CI	p-value	Effect size
	Mean (SD) [Range]		Mean (SD) [Range]			
Assumed sleep time	08:26 (00:59) [06:32–10:16]	[07:59,08:53]	07:39 (01:02) [05:22–08:35]	[06:47,08:31]	.15ˆ	.57
Actual sleep time	07:10 (00:53) [05:25–09:20]	[06:45,07:34]	06:56 (00:55) [04:58–07:54]	[06:09,07:43]	.56	.25
Sleep efficiency (%)	69.27 (14.85) [28.75–85.60]	[62.52,76.04]	77.38 (10.29) [54.00–86.50]	[68.77,85.98]	.06ˆ	.76
Lights out	21:06 (01:00) [19:25–03:33]	[20:38,21:34]	22:41 (00:38) [21:30–23:22]	[22:09,23:13]	<.001${}^{***}$	−1.70
Fell asleep	22:07 (01:06) [19:49–23:59]	[21:17,22:37]	23:22 (00:47) [22:40–01:48]	[22:53,00:12]	.001${}^{***}$	−1.38
Sleep onset latency (min)	65 (40) [3–147]	[47,84]	48 (49) [15–149]	[6,89]	.15ˆ	.56
Waketime	06:49 (00:45) [05:51–08:52]	[06:28,07:10]	07:24 (00:33) [06:23–07:56]	[06:56,07:51]	.04${}^{*}$	−.80
Get up	07:12 (00:46) [05:51–09:18]	[06:51,07:33]	07:41 (00:35) [06:52–08:22]	[07:11,08:10]	.09	−.66
Time in bed	09:50 (01:06) [07:45–12:34]	[09:19,10:20]	08:53 (00:48) [07:29–10:28]	[08:12,09:33]	.21	.91
Wake bout duration (min)	1 (0) [0–2]	[1,2]	3 (5) [1–16]	[0,8]	.03∗,^ˆ	.12
Wake bout frequency (no.)	38.66 (12.25) [17.00–67.80]	[33.08,44.23]	37.66 (10.50) [18.00–49.50]	[28.89,46.46]	.83	.08
Fragmentation index (%)	26.39 (8.37) [13.40–40.70]	[22.58,30.21]	23.88 (4.51) [15.60–29.20]	[20.11,27.64]	.31	.33

Unless indicated otherwise, all units are in terms of time (HH:MM). Note that “∧” indicates non-parametric test used in the comparison between mother and child data, while “${}^{*}$” Indicates statistical significance.

Analysing mothers’ actigraphy data according to their child’s corresponding age group (Table 5), no statistically significant results emerged. However, to briefly summarise the findings of this analysis, mothers of school-aged children slept on average 19 min less with mean sleep duration of 6 h and 25 min (SD=57 min) compared to mothers of adolescent aged children, with sleep duration of 6 h and 44 min (SD=1 h 3 min). At the same time, mothers of adolescents with ASD fell asleep approximately 44 min later (SD=1 h 29 min) and experienced longer night awakenings (mean=9 min, SD=13 min) compared to mothers of school-aged autistic children (average sleep onset time at 2,350, SD=1 h 6 min; mean duration of night awakenings = 2 min, SD=0 min).

**Table 5:** Sleep profiles of mothers according to their child’s age group based on actigraphy and sleep diary data.

Actigraphy variable	Mothers of school-aged child	Mothers of child 95%CI	Mothers of adolescent	Mothers of adolescent 95%CI	p-value	Effect size
	Mean (SD) [Range]		Mean (SD) [Range]			
Assumed sleep time	07:34 (01:01) [05:01–09:28]	[07:06,08:02]	07:35 (00:53) [06:11–08:59]	[06:47,08:31]	.96	−.02
Actual sleep time	06:25 (00:57) [05:25–09:20]	[05:59,06:52]	06:44 (01:03) [05:30–08:48]	[05:51,07:37]	.48	−.32
Sleep efficiency (%)	79.30 (5.83) [68.80–87.60]	[75.64,81.96]	80.32 (7.62) [71.00–89.90]	[73.95,86.68]	.74	−.16
Lights out	23:25 (01:12) [21:17–01:42]	[22:52,23:58]	00:08 (01:36) [22:22–03:34]	[22:47,01:28]	.28	−.54
Fell asleep	23:50 (01:06) [22:03–01:53]	[23:19,00:20]	00:34 (01:29) [23:19–01:48]	[23:10,03:46]	.23	−.6
Sleep onset latency (min)	21 (13) [10–53]	[15,27]	25 (49) [12–50]	[15,35]	.26ˆ	.44
Waketime	07:16 (00:56) [06:10–10:44]	[06:51,07:42]	07:39 (01:18) [06:38–10:49]	[06:33,08:54]	.58ˆ	.21
Get up	07:22 (00:57) [06:20–10:49]	[06:56,07:48]	08:00 (01:15) [06:52–10:50]	[06:57,09:03]	.15ˆ	.57
Time in bed	07:57 (01:01) [05:11–09:50]	[07:29,08:26]	08:02 (00:48) [06:48–09:16]	[07:18,08:46]	.84	−.08
Wake bout duration (min)	2 (0) [1–4]	[1,2]	9 (13) [0–40]	[−1,21]	.35ˆ	.37
Wake bout frequency (no.)	37.66 (11.97) [17.80–78.80]	[32.21,43.11]	39.10 (21.52) [20–85.10]	[18.10,54.10]	.28ˆ	.43
Fragmentation index (%)	26.73 (8.66) [13.70–47.60]	[22.70,30.77]	27.00 (4.51) [15.00–46.70]	[18.91,35.10]	.95	−.03

Unless indicated otherwise, all units are in terms of time (HH:MM). Note that “∧” indicates non-parametric test used in the comparison between mother and child data.

### 3.3. Relationship between cortisol and actigraphy

Prior to group analysis, data from one mother whose cortisol samples showed unusually high levels was removed. Average maternal morning cortisol was 5.43 ng/ml (SD=5.25, range=.57-22.82), with a diurnal cortisol ratio of .54 (SD=.51; range=.03-2.21). Therefore, there were large individual variabilities in cortisol expression within the present sample. As normative cortisol values are not established, and it is unknown if aging or individuals from different age groups would express significantly different levels of cortisol over time, the focus of the present analysis is on the daily patterns of cortisol expression within the current sample and not comparisons with a population norm.

Only Sleep Onset Latency correlated significantly with morning cortisol (r(29)=.38, p=.045). All other correlations reached non-significance after controlling for age.

Comparing by cortisol profiles, although not significant, mothers with a flattened cortisol profile (i.e., below the sample mean; n=18) experienced more night awakenings (mean=5, SD=9, ranging from 1 to 40), compared to mothers with a normal cortisol profile (i.e., above the sample mean; n=114; mean=1, SD=0, ranging from 0 to 4; p=.10, d=.65; Table 6).

**Table 6:** Sleep profiles of mothers according to their cortisol profiles based on actigraphy and sleep diary data.

Actigraphy variable	Normal cortisol profile	Normal cortisol 95%CI	Flattened cortisol profile	Flattened cortisol 95%CI	p-value	Effect size
	Mean (SD) [Range]		Mean (SD) [Range]			
Assumed sleep time	07:29 (00:51) [06:06–08:48]	[07:00,07:59]	07:38 (01:04) [05:01–09:28]	[07:08,08:07]	.73	−.13
Actual sleep time	06:28 (00:52) [04:47–07:31]	[05:53,07:03]	06:32 (01:03) [03:53–08:48]	[06:01,07:04]	.84	.07
Sleep efficiency (%)	77.83 (6.25) [68.80–85.80]	[73.63,82.03]	80.66 (6.17) [69.70–89.90]	[77.59,83.73]	.25	.46
Lights Out	23:38 (01:40) [21:42–03:34]	[22:31,00:45]	23:36 (01:09) [21:17–01:42]	[21:06,21:59]	.96	−.02
Fell asleep	00:07 (01:35) [22:03–03:46]	[23:03,01:11]	22:59 (01:01) [22:03–01:53]	[23:28,00:30]	.81	−.11
Sleep onset latency (min)	25 (13) [10–50]	[16,34]	21 (12) [10–53]	[15,27]	.30ˆ	.40
Waketime	07:33 (01:10) [06:24–10:49]	[06:46,08:21]	07:16 (00:58) [06:10–10:44]	[06:47,07:45]	.2ˆ	.49
Get up	07:41 (01:09) [06:28–10:50]	[06:54,08:27]	07:28 (01:01) [06:20–10:49]	[06:57,07:58]	.44ˆ	.30
Time in Bed	07:59 (00:48) [06:32–09:00]	[07:26,08:32]	07:58 (01:05) [05:11–09:50]	[07:26,08:31]	.97	−.01
Wake bout duration (min)	1 (0) [0–4]	[1,2]	5 (9) [1–40]	[0,10]	.10ˆ	.65
Wake bout frequency (no.)	42.32 (21.15) [21.00–85.10]	[31.69,42.86]	34.12 (8.37) [17.80–46.00]	[29.96,38.28]	.47ˆ	.29
Fragmentation index (%)	26.78 (6.00) [15.40–38.50]	[22.74,30.81]	26.83 (10.48) [13.70–47.60]	[21.62,32.04]	.99	.01

Unless indicated otherwise, all units are in terms of time (HH:MM). Note that “∧” indicates non-parametric test used in the comparison between mother and child data.

### 3.4. Hierarchical multiple regression

#### 3.4.1. Predictors of total sleep time in children

In step one, age and sex of the child, and the child’s CARS-2 score were entered into the model as control variables. The second step added sleep variables from actigraphy (i.e., sleep onset latency and time spent in bed). CSHQ score was added at step 3. The regression model showed that age, sex and CARS-2 did not significantly contribute to the model at step one, F(1,28)=.94, p=.44. Adding sleep variables at step two revealed that sleep onset latency and time spent in bed significantly contribute to the total sleep time in children with ASD, F(1,28)=11.90, p<.001, adjusted $R^{2}=.72$ (see Table 7). The final model was significant with sleep onset latency, time in bed and CSHQ score predicting total sleep time in children, F(1,28)=13.29, p<.001, adjusted $R^{2}=.78$. The model found that total sleep time tends to increase with more time spent in bed, and lower sleep onset latency and CSHQ score.

**Table 7:** Hierarchical multiple regression predicting Actual Sleep Time in children.

Step	Variable	β	p-value	Adjusted $R^{2}$
1	Age of child	−.18	.36	10
	Child’s sex	−.26	.22	
	CARS-2 score	−.07	.74	
2	Age of child	.2	.14	.72${}^{**}$
	Child’s sex	.08	.50	
	CARS-2 score	−.06	.63	
	Child’s sleep onset latency	.82	<.001	
	Child’s time in bed	1.11	<.001	
3	Age of child	.18	.15	.78${}^{**}$
	Child’s sex	.08	.49	
	CARS-2 score	−.003	.98	
	Child’s sleep onset latency	−.74	<.001	
	Child’s time in bed	1.01	<.001	
	CSHQ score	−.27	.02	

#### 3.4.2. Predictors of total sleep time in mothers

Step one involved entering mothers’ age and their child’s age, as well as CARS-2 score as a control variable. The second step added sleep variables from actigraphy (i.e., sleep onset latency, time spent in bed and fragmentation index). The third step added the child’s sleep variables (i.e., number of wake bouts and time spent in bed). The regression model showed that mothers’ age, their child’s age and CARS-2 score did not significantly contribute to the model at step one, F(1,28)=.77, p=.52. Adding mothers’ sleep variables at step two revealed that child age, sleep onset latency, time in bed and fragmentation index are significantly contributing to the model, F(1,28)=31.03, p<.001, adjusted $R^{2}=.87$. The third step revealed that the full model of mothers’ sleep onset latency, time in bed, fragmentation index, child’s age and time in bed contribute significantly to mothers’ total sleep time, F(1,28)=28.24, p<.001, adjusted $R^{2}=.89$, (see Table 8). The model found that total sleep time of mothers tends to increase with more time spent in bed, lower sleep onset latency and fragmentation index, as well as with an older child and who spends more time in bed.

**Table 8:** Hierarchical multiple regression predicting Actual Sleep Time in mothers.

Step	Variable	β	p-value	Adjusted $R^{2}$
1	Age of mother	−.22	.37	.08
	Age of child	.35	.16	
	CARS-2 score	−.12	.54	
2	Age of mother	−.16	.13	.89${}^{**}$
	Age of child	.30	.004	
	CARS-2 score	.03	.68	
	Mother’s sleep onset latency	−.30	<.001	
	Mother’s time in bed	.91	<.001	
	Mother’s fragmentation index	−.36	<.001	
3	Age of mother	−.16	.11	.92${}^{**}$
	Age of child	.39	<.001	
	CARS-2 score	.02	.31	
	Mother’s sleep onset latency	−.29	<.001	
	Mother’s time in bed	.95	<.001	
	Mother’s fragmentation index	−.34	<.001	
	Child’s time in bed	.17	.041	
	Child’s wake bout frequency	−.11	.13	

#### 3.4.3. Predictors of sleep fragmentation in mothers

Step one entered mothers’ age, their child’s age and CARS score as a control variable. The second step added sleep variables from actigraphy (i.e., sleep onset latency and sleep efficiency) and wake up time. The third step added the child’s sleep variables (i.e., CSHQ sleep disordered breathing and night waking). The regression model showed that only mothers’ age significantly contributed to the model at step one, F(1,28)=1.98, p=.14. Adding mothers’ sleep variables at step two revealed that sleep efficiency and wake up time significantly contributed to the model, F(1,28)=4.82, p=.003, adjusted $R^{2}=.45$. The third step revealed that the full model of mothers’ sleep efficiency, sleep onset latency, wake up time and their child’s sleep disordered breathing contribute significantly to mothers’ fragmented sleep, F(1,28)=5.19, p=.001, adjusted $R^{2}=.55$ (see Table 9). The model found that the fragmentation index of mothers tends to decrease with better sleep efficiency, and longer sleep onset latency. Fragmentation index tends to increase for mothers with later wake up times and a child who shows more symptoms of sleep disordered breathing.

**Table 9:** Hierarchical multiple regression predicting Fragmentation Index in mothers.

Step	Variable	β	p-value	Adjusted $R^{2}$
1	Age of mother	.53	.031	.2
	Age of child	−.43	.068	
	CARS-2 score	.17	.37	
2	Age of mother	.14	.5	.57${}^{*}$
	Age of child	−.10	.63	
	CARS-2 score	.18	.22	
	Mother’s sleep onset latency	−.32	.09	
	Mother’s sleep efficiency	−.55	.01	
	Mother’s waketime	.41	.01	
3	Age of mother	.09	.64	.68${}^{**}$
	Age of child	−.07	.72	
	CARS-2 score	.2	.2	
	Mother’s sleep onset latency	−.50	.02	
	Mother’s sleep efficiency	−.56	.003	
	Mother’s waketime	.53	.001	
	Child’s sleep disordered breathing	.46	.021	
	Child’s wake bout frequency	−.25	.24	

#### 3.4.4. Predictors of night wakings in mothers

Step one involved the entering of mothers’ age and their child’s age, sex of child and CARS-2 score as a control variable. The second step added the child’s mean duration of night wakings, as measured from actigraphy. The regression model showed that child’s age and the female child sex significantly contribute to the model at step one, F(1,28)=2.93, p=.042, adjusted $R^{2}=.22$. Adding child’s night waking at step two revealed that the full model of child’s age and night waking contribute significantly to mothers’ night wakings, F(1,28)=11.32, p<.001, adjusted $R^{2}=.65$, (see Table 10). The model found that mothers experiencing longer night wakings with an older child and longer night wakings of the child.

**Table 10:** Hierarchical multiple regression predicting Wake Bout Duration in mothers.

Step	Variable	β	p-value	Adjusted $R^{2}$
1	Age of mother	−.19	.37	.33${}^{*}$
	Age of child	.54	.02	
	Child’s sex	.46	.02	
	CARS-2 score	−.13	.47	
2	Age of mother	−.15	.29	.71${}^{**}$
	Age of child	.37	.02	
	Child’s sex	.2	.14	
	CARS-2 score	−.19	.13	
	Child’s wake bout duration	.69	<.0001	

#### 3.4.5. Predictors of morning cortisol in mothers

Step one involved entering mothers’ age and their child’s age and CARS-2 score as a control variable. The second step added mothers’ diurnal cortisol ratio. The third step added mothers’ wake bouts at night followed by the child’s CSHQ total score at step four. At step one the regression model showed no significance, F(1,28)=1.44, p=.15. Adding diurnal cortisol ratio at step two revealed that the model was significant, F(1,28)=4.90, p=.005, adjusted $R^{2}=.45$ (see Table 11). The full model was significant, with diurnal cortisol ratio, mothers’ wake bouts and child’s CSHQ score predicting maternal morning cortisol (F(1,28)=7.23, p<.001, adjusted $R^{2}=.66$). The model found that morning cortisol tends to be high for mothers with lower diurnal cortisol ratio but greater wake bouts at night. Morning cortisol appeared to be lower for mothers with a child who had a higher CSHQ score, thus indicating more severe sleep problems.

**Table 11:** Hierarchical multiple regression predicting morning cortisol in mothers.

Step	Variable	β	p-value	Adjusted $R^{2}$
1	Age of mother	.25	.31	.15
	Age of child	−.42	.09	
	CARS-2 score	−.16	.39	
2	Age of mother	.11	.6	.45${}^{*}$
	Age of child	−.41	.04	
	CARS-2 score	−.11	.5	
	Diurnal cortisol ratio	−.57	.001	
3	Age of mother	.09	.64	.56${}^{**}$
	Age of child	−.07	.72	
	CARS-2 score	.2	.2	
	Diurnal cortisol ratio	−.50	.02	
	Mother’s wake bout frequency	−.56	.003	
4	Age of mother	.26	.88	.66${}^{**}$
	Age of child	−.27	.12	
	CARS-2 score	.03	.81	
	Diurnal cortisol ratio	−.59	<.001	
	Mother’s wake bout frequency	.42	.01	
	CSHQ score	−.34	.02	

### 3.5. Dyadic mother-child analysis of total sleep time

**Figure 1:**
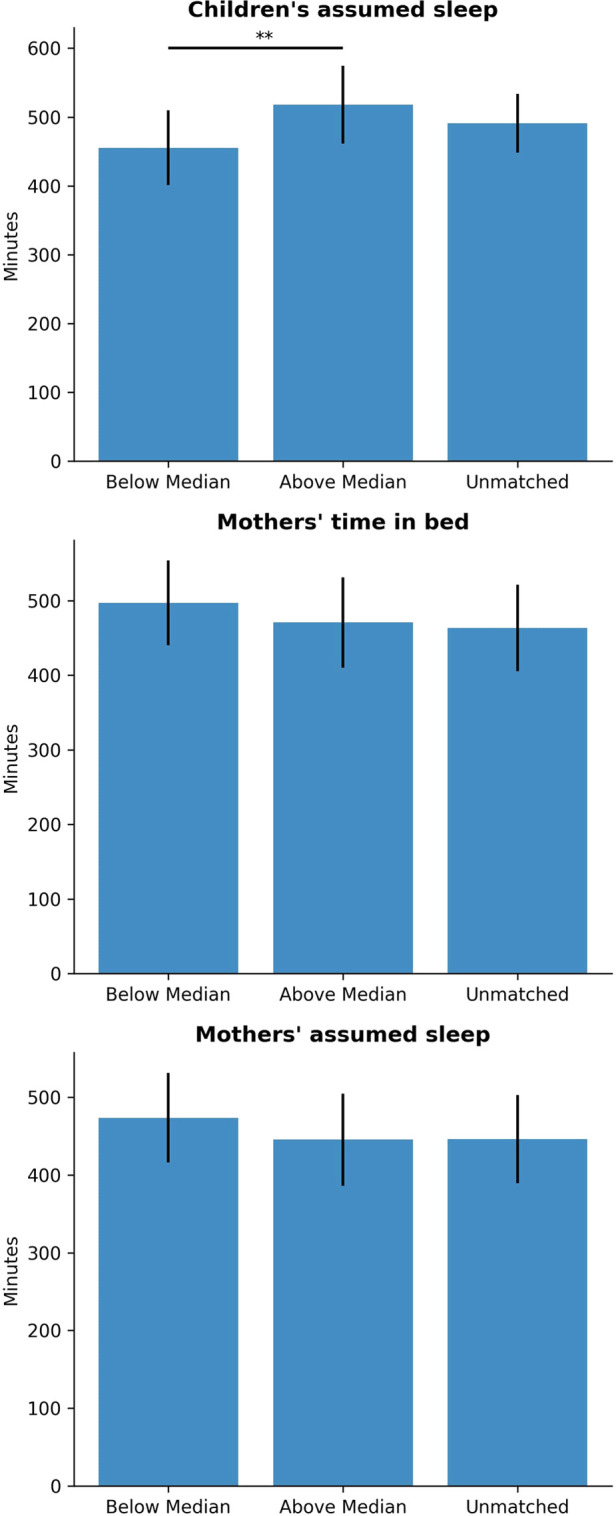
Comparison of sleep variables by group (matching of mother-child sleep). ${}^{*}p<.05$, ${}^{**}p<.0014$.

Lastly, we conducted an exploratory analysis of mothers’ and children’s sleeping time. Mother-child dyads were classified according to their actual sleep time with reference to the median value of the variable. If both the mother and child were on the lower part of the median, the pair was classified as “Below Median,” while if both the mother and children were on the higher part of the median, the pair was classified as “Above Median.” In the case in which the mother and the children were not on the same part of the distribution (e.g., child actual sleep time below the median, while mother sleep time above the median), the pair was classified as “Unmatched.” Multiple F tests (one-way ANOVA) were conducted to make comparisons these independent groups of mother-child dyads for different dependent variables: (1) Children’s assumed sleep, (2) Mothers’ time in bed, (3) Mothers’ assumed sleep. For each variable, one F test was conducted with post-hoc t-tests if results were statistically significant. Significant differences were found with respect to children’s assumed sleep (F(1,28)=5.791, p=.001), mothers’ time in bed (F(1,28)=7.557, p=.002), and mothers’ assumed sleep (F(1,28)=6.167, p=.006; see Figure 1). Post-hoc t-tests revealed that there are significant differences between “Above Median” and “Below Median” groups in children’s assumed sleep (t=−2.84, p=0.00846), but no other significant between-groups differences for mothers’ time in bed and assumed sleep.

Next, the distribution of the variables with respect to absolute differences in children’s and mothers’ Actual Sleep Time were compared. The difference in Actual Sleep Time significantly correlates with children’s assumed sleep (r=.488, p=.007), mothers’ time of falling asleep (r=−.375, p=.045), mothers’ time in bed (r=−.532, p=.002), and mothers’ assumed sleep time (r=−.516, p=.004). In other words, the more disparate the mother’s and child’s Actual Sleep Time, the later the mothers’ Fall Asleep time and Assumed Sleep Time, and the shorter the mothers’ Time in Bed. Conversely, the greater the difference between the mother’s and child’s Actual Sleep Time, the greater the child’s Assumed Sleep Time, which may be point to the fact that despite the analysis of a directionally-neutral absolute difference in Actual Sleep Time, the discrepancy between mother and child Actual Sleep Time are related to longer child sleep and a shorter duration of mother sleep.

**Figure 2:**
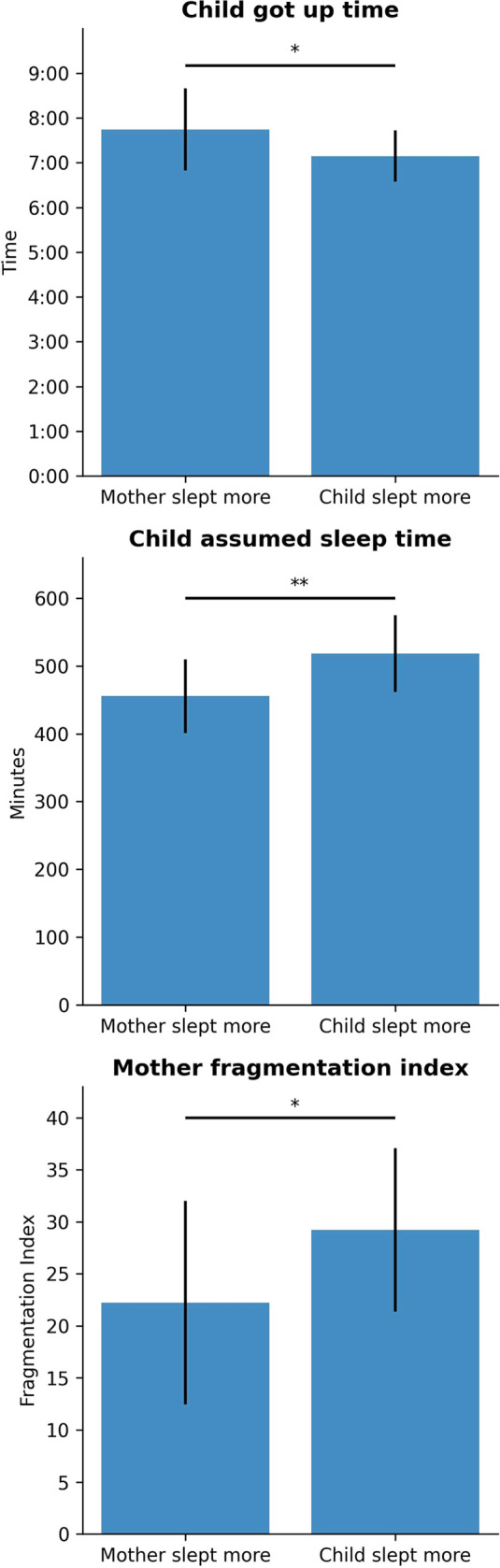
Comparison of sleep variables by group (mother slept more or child slept more). ${}^{*}p<.05$, ${}^{**}p<.001$.

Subsequently, the pair of mother and children was divided in two groups: groups in which the mother slept (actual sleep time) more than the children, and groups in which the children actual sleep time was higher than the mothers’. Significant differences between the groups were found for children’s getting up time (t=2.126, p=.042), children’s assumed sleep time (t=−2.849, p=.008), and mothers’ fragmentation index (t=−2.077, p=.047; Figure 2).

## 4. Discussion

The present study set out to explore, using both mother- and child-related variables, significant predictors of sleep quality in both mothers and their children with ASD by collecting data from both subjective and objective sources. To this end, the analysis followed four main statistical approaches: (1) initial descriptive and comparative results from actigraphy and sleep diary data, (2) correlation between cortisol and actigraphy data, (3) exploratory hierarchical multiple regressions, and (4) dyadic analysis.

Before discussing the results of the main statistical approaches, ASD characteristics of the recruited sample are interpreted using clinical cutoffs for CARS-2 (45). A mean score of 39 indicated severe signs of ASD, with the lowest still meeting the clinical cutoff for ASD diagnosis (45). Overall, child participants would be classified as exhibiting moderate to severe signs of ASD by their mothers.

Firstly, based on the findings from descriptive actigraphy data, it was found that mothers slept approximately 6.5 h every night on average, which is slightly below the optimum 7 to 9 h of sleep for adults recommended by National Sleep Foundation (51). Generally, mothers tended to sleep significantly less compared to their child, which aligns with common understanding that caregivers often rest only after their child is asleep. A closer inspection of actigraphy data, however, also revealed a large variability in sleep duration, where some mothers slept only 5 h a night, while others slept over 9 h. This variation in sleep duration was not modulated by the different age groups of their child with ASD, although causality cannot be confirmed using the present cross-sectional research design. Nonetheless, the findings here lend the opportunity to discuss the strengths of adopting both subjective and objective measures in the study design. While there was concordance between actigraphy data and sleep diary data in terms of sleep duration, it was found that mothers tended to estimate their sleep onset times to be earlier than their actual sleep onset. Actigraphy data revealed that mothers had sleep onset delay, taking up to an hour to fall asleep (average value in the present study is 20 min). Taken together, these findings lend support to current literature that mothers of children with ASD are chronically sleep-deprived and have poorer psychological functioning (52, 53).

On the actigraphy measure, children showed similar sleep deprivation as reported previously by Hering and colleagues (54) with sleep duration less than 8 h. Children in the current study, regardless of age, had sleep duration of approximately 7 h. They also showed prolonged sleep onset of over one hour and frequent wake bouts. When comparing actigraphy data across the different age groups, it was found that adolescents tended to fall asleep later than school-going children, which is consistent with developmental literature on adolescents experiencing delayed circadian rhythms (55, 56). However, it must be noted that unlike the developmental trajectories of typically developing children (5, 8, 6, 7), we did not observe the same reduction in sleep problems in the adolescent group; in fact, the duration of wake bouts in the adolescent group is significantly longer as compared to school-going children. This finding may lend support to the potential worsening of sleep-related problems among individuals with ASD as they age, that was not successfully mitigated by the enforcing of regular schedules. It is still not yet known why sleep problems may worsen over time among children with ASD, although it may have to do with unresolved problems with arousal and anxiety that is commonly associated with ASD (57–59). These common co-morbidities of ASD contribute to hyperarousal due to heightened sensitivity, which may contribute to greater difficulties in falling back asleep (60, 61), although further studies would be needed to confirm this conjecture as it is unknown if the present sample of children had other undiagnosed co-morbidities. Nonetheless, descriptive findings from actigraphy data point to the need for a developmental perspective when considering habilitation for sleep problems in children with ASD. It may be insufficient to assume that regular daytime schedules would be helpful in alleviating some of the sleep-related issues faced by these children. Supporting a conducive sleeping environment for adolescents (e.g., by limiting screentime and caffeine intake (62)) may also be of importance. To this end, studies by McLay and colleagues (63) and Van and colleagues (64) have designed and evaluated functional and behavioural interventions respectively to aid sleep problems among adolescents with ASD, with some success.

More generally, according to the comparative findings, there are numerous differences in terms of the quantity of mother and child sleep. This was particularly evidenced by significant differences in sleep efficiency, time in bed and sleep onset latency. There are two plausible suggestions put forward here. One is related to the age of the child; as all the children in the study were of schooling age, parents have had a substantial number of years to become more familiar and confident about their child’s sleep habits and safety without needing to attend to them at night. Also, the sleeping environment, a factor that was not explored in this study, could have been modified to make the bedroom safe for the child. These developments may lead to more flexibility in dictating personal sleeping and resting hours, therefore reducing group similarities between mother and child. Additionally, older children might have learnt not to call for parental attention at night but instead learnt to engage in self-soothing or regulatory activities to fall back asleep. While this may be interpreted as an encouraging sign that children with ASD may form independent sleep patterns, it must still be noted that both mother and child actigraphy data show a slew of sleep-related problems.

In the second analytical approach, variables from the two objective measures: cortisol and actigraphy were correlated with each other. Findings showed that greater sleep onset latency was related to higher amounts of morning cortisol, which contradicted past studies that found negative correlations between sleep disturbances or sleep restriction and morning cortisol (65, 66). A similar study on morning cortisol and sleep duration in children found the same negative correlation between the two (67), which may indicate that our finding should be interpreted with caution. It should be noted that the significance level of this correlation was not high; in fact, it is only .045. However, it is also worth mentioning that previous studies did not use actigraphy as a measure of sleep behaviour, but rather self-reported questionnaires (66) and experimental manipulation of sleep duration (65). Another finding within this approach is that a flattened cortisol profile was correlated with more frequent wake bouts, which is more aligned with the previously established literature (65, 66), although this was not statistically significant. Taken together, it appears that further research is needed to verify the directionality and effect sizes of the relationship between sleep and cortisol. Nonetheless, the present findings add to the current literature that poor sleep can contribute to physiological changes within the individual (34–37), although caution should be taken that the current findings are correlational in nature.

The third set of findings pertained to exploratory hierarchical multiple regressions. It was revealed that a variety of mother- and child-related variables were predictive of sleep duration and quality in both mother and child. Particularly, time in bed emerged as a significant predictor of sleep quantity (i.e., total sleep time) for both mother and child. Sleep onset latency has also emerged as a significant predictor for both mother and child’s sleep quantity, as well as sleep quality (i.e., sleep fragmentation and wake bout duration) in mothers, thus adding to its robustness as an indicator of sleep. Some evidence of bi-directional influence of sleep has also emerged, where the child’s sleep disordered breathing and wake bout duration have been found to be significant predictors of mothers’ sleep fragmentation and wake bout duration respectively. Similar findings have been found, where the child’s sleep problems are related to the mothers’ reports of sleep issues (68, 69), and may be because the child’s sleep disturbances tended to also awaken their caregivers (70, 69), although this is less likely if the child and mother sleep in different rooms (thereby not alerting the mothers). Unfortunately, information on sleeping arrangements was not obtained in the present study, and may be considered for future studies interested in this area of mother-child sleep. Additionally, significant predictors of morning cortisol in mothers were also uncovered. Interestingly, frequency of wake bouts was found to be significant predictors of morning cortisol, which may point to the effect of fragmented sleep on stress (71). However, similar to the above, hierarchical regression analyses are also correlational and predictors may not necessarily indicate causality. Future studies may wish to verify the directionality and causality of these relationships. Family systems-oriented interventions should consider the impact of poor sleep on caregivers and may wish to involve other family members in the care of the child with ASD, as is also recommended by (72).

In the last analytic approach, the mother-child dyad variable was created to determine if there are group differences between mothers and their children with ASD who show similar or dissimilar sleep time. However, no post-hoc results were significant between matched and unmatched groups. It appears that similar sleep patterns between mother and child may not be significant in eventual sleep quantity or quality of mother or child. Next, larger differences in actual sleep time between mother and child were found to be correlated with more assumed sleep time for the child, and a shorter time in bed for the mothers. These findings may point to the tendency to over-report sleep times. Finally, in groups where the mothers slept more than the child, their children got up significantly later than their peers whose mothers slept less. Mothers who slept more than the child also experienced significantly lower rates of sleep fragmentation than if their child slept more. These results point to potential interesting effects of mother sleep where a greater duration of sleep by the mothers could lead to both improved sleep quantity for their child and sleep quality for themselves, and may be in support of the fact that parental well-being is of utmost importance when considering habilitation for sleep-related problems in ASD (1, 15). Of course, as causality cannot be ascertained in the present research design, other than the above interpretation, another simple explanation may be that mothers who have less fragmented sleep naturally have a higher likelihood of sleeping more than their child, with all other factors remaining equal. Nonetheless, in evaluating the parental and familial components of a habilitation or intervention, Karst and Van Hecke (72) recommends a model based on these bi-directional transactions between parent and child to account for the well-being of these important stakeholders in the child’s treatment process, although research in this particular area is still sparse.

### 4.1. Study evaluation and future recommendations

In contrast to a past study reporting sensory hypersensitivity and data loss due to poor adherence among children with ASD (73), this study did not find significant data loss among our sample. To mitigate this potential issue, children in the current study had been monitored for signs of discomfort, and the standard actigraphy straps had been replaced with hospital bands to reduce hypersensitivity while wearing the device.

Several future studies, clinical recommendations and suggestions have been mentioned throughout the discussion above. To summarise, we encourage practitioners and policy makers to take into account caregiving burden and caregiver needs, particularly from a sleep perspective, when formulating interventions. Secondly, the integration of sleep education and existing behavioural interventions for children with ASD may also be helpful in improving ASD prognosis and family sleep. Thirdly, we recommend further research into determining the causality of various sleep behaviours on mother and child wellbeing, taking into account environmental and other contextual factors such as sleep arrangements and lifestyle choices to improve the applicability of our current models and enhance current understanding of family systems in the area of sleep.

However, a prominent limitation of this study is in its small sample size, especially as the analysis had further segmented the children by age into school-going children and adolescents. Additionally, as a cross-sectional design, causality cannot be determined, particularly in the developmental trajectories of sleep behaviour in children with ASD. Nevertheless, the study design has strengths in terms of adopting both subjective and objective sleep measures, as well as for proposing novel dyadic analyses in examining mother-child sleep.

Future studies may consider replicating this study in other countries where sleep environments and arrangements are different (e.g., examining co-sleeping arrangements) to extend the external validity of the present findings, as well as adopt a larger sample size that includes a control (typically developing) group. Additionally, other lifestyle factors (e.g., nighttime blue light exposure, caffeine use, bedtime routines) and family structures (e.g., co-parenting, grandparents or other family relatives, siblings) can be included to provide more ecological validity.

## 5. Conclusion

The present study aimed to explore significant predictors of sleep in mothers and their children with ASD by using a mixture of objective and subjective measures. From the above findings, it emerged that both mother and child were likely to experience sleep deprivation in terms of shorter sleep duration. Additionally, they were also likely to experience poorer sleep quality, as shown by the longer sleep onset latencies and higher frequencies of wake bouts. Findings also showed that the child’s sleep behaviours (e.g., presence of sleep disordered breathing, duration of wake bouts) have a significant impact on the quality of mothers’ sleep, providing initial evidence of the interaction between mothers’ and children’s sleep habits.

## Data Availability

The raw data supporting the conclusions of this article will be made available by the authors, without undue reservation.
